# The Association between Fatal Coronary Heart Disease and Ambient Particulate Air Pollution: Are Females at Greater Risk?

**DOI:** 10.1289/ehp.8190

**Published:** 2005-08-02

**Authors:** Lie Hong Chen, Synnove F. Knutsen, David Shavlik, W. Lawrence Beeson, Floyd Petersen, Mark Ghamsary, David Abbey

**Affiliations:** Department of Epidemiology and Biostatistics, Loma Linda University, Loma Linda, California, USA

**Keywords:** air pollution, coronary disease, ischemic heart disease, long-term exposure, mortality, particulate matter

## Abstract

The purpose of this study was to assess the effect of long-term ambient particulate matter (PM) on risk of fatal coronary heart disease (CHD). A cohort of 3,239 nonsmoking, non-Hispanic white adults was followed for 22 years. Monthly concentrations of ambient air pollutants were obtained from monitoring stations [PM < 10 μm in aerodynamic diameter (PM_10_), ozone, sulfur dioxide, nitrogen dioxide] or airport visibility data [PM < 2.5 μm in aerodynamic diameter (PM_2.5_)] and interpolated to ZIP code centroids of work and residence locations. All participants had completed a detailed lifestyle questionnaire at baseline (1976), and follow-up information on environmental tobacco smoke and other personal sources of air pollution were available from four subsequent questionnaires from 1977 through 2000. Persons with prevalent CHD, stroke, or diabetes at baseline (1976) were excluded, and analyses were controlled for a number of potential confounders, including lifestyle. In females, the relative risk (RR) for fatal CHD with each 10-μg/m^3^ increase in PM_2.5_ was 1.42 [95% confidence interval (CI), 1.06–1.90] in the single-pollutant model and 2.00 (95% CI, 1.51–2.64) in the two-pollutant model with O_3_. Corresponding RRs for a 10-μg/m^3^ increase in PM_10-2.5_ and PM_10_ were 1.62 and 1.45, respectively, in all females and 1.85 and 1.52 in postmenopausal females. No associations were found in males. A positive association with fatal CHD was found with all three PM fractions in females but not in males. The risk estimates were strengthened when adjusting for gaseous pollutants, especially O_3_, and were highest for PM_2.5_. These findings could have great implications for policy regulations.

Since the early reports of increased deaths from cardiopulmonary disease (CPD) after serious air pollution episodes (Firket 1931; Logan 1953), studies both within the United States and abroad have found similar short-term effects of air pollution (Dominici et al. 2003; Samet et al. 2000; Zanobetti et al. 2003).

Studies have also found increased risk of CPD, noncancer respiratory, and respiratory cancer deaths with chronic exposure to ambient particulate matter (PM) (Abbey et al. 1999; Dockery et al. 1993; McDonnell et al. 2000; Pope et al. 1995, 2002, 2004a), black smoke (NO_x_) (Hoek et al. 2002), and nitrogen oxides (Hoek et al. 2002; Nafstad et al. 2004). Four main prospective studies have been conducted in the United States to assess long-term health effects of ambient air pollution in adults [the Six Cities Study, the American Cancer Society (ACS) study, the Adventist Health Study on the Health Effects of Smog (AHSMOG), and the national cohort of male U.S. veterans]. Associations with fine particulates [PM < 2.5 μm in aero-dynamic diameter (PM_2.5_)] have been found for all-cause mortality, CPD mortality, and respiratory/lung cancer mortality in the ACS, Six Cities, and AHSMOG studies and with mortality attributable to ischemic heart disease (IHD), dysrhythmias, heart failure, and cardiac arrest in the ACS study. AHSMOG (Abbey et al. 1999) has also shown positive associations, although not always significant, between PM < 10 μm in aerodynamic diameter (PM_10_) and all-natural-cause mortality and CPD mortality in males but not in females. For fatal lung cancer and any mention of non-malignant respiratory disease, a positive association was found with PM_10_ in both sexes. The national cohort of male U.S. veterans, where all subjects were hypertensive at baseline, found no increased mortality with increasing levels of fine particulates (Lipfert et al. 2000). From Europe, Hoek et al. (2002) reported increased risk of CPD mortality and all-cause mortality with increased concentrations of black smoke and nitrogen dioxide, and Nafstad et al. (2004) found increased risk of noncancer respiratory mortality and CPD mortality with increasing levels of NO_x_.

Several studies on short-term effects have found that ambient PM increases cardiac arrhythmia (Peters et al. 2000), decreases heart rate variability (Pope et al. 2004b), increases the inflammatory response measured by C-reactive protein (CRP) (Riediker et al. 2004), and increases blood viscosity (Peters et al. 1997) as well as other blood markers (e.g., hemoglobin, fibrinogen, platelet counts, white cell counts) (Riediker et al. 2004). These observed effects would provide a mechanism by which chronic exposure to ambient air pollution is associated with risk of coronary heart disease (CHD).

This study reports on the risk of fatal CHD associated with long-term ambient air pollution in AHSMOG.

## Materials and Methods

### Study population.

AHSMOG began in April 1977 by enrolling 6,338 participants from the Adventist Health Study (AHS) (*n* = 34,198), a large cohort study of the relationship between lifestyle and risk of chronic disease (Beeson et al. 1989). To be included in AHSMOG, subjects must be nonsmoking, non-Hispanic whites ≥25 years of age at baseline and must have lived ≥10 years within 5 miles of their 1976 neighborhood. All subjects satisfying these criteria were selected from three large metropolitan areas in California: San Francisco, South Coast (i.e., Los Angeles and eastward), and San Diego air basins. In addition, a 13% random sample of 862 AHS subjects was selected from the rest of California assuring large variation and wide ranges in concentrations of different ambient air pollutants.

As part of their enrollment in the AHS in 1976, all participants completed a comprehensive questionnaire that included questions on education, anthropometric data, smoking history, dietary habits, exercise patterns, and previous physician-diagnosed chronic diseases (Beeson et al. 1989). Monthly residence and work location histories were obtained for each subject for the period January 1966 through December 1998, or until date of death or date of last contact, by using mailed questionnaires (1977, 1987, 1992, 2000), tracing by telephone, and interviewing of surrogates (for deceased subjects). Only 29 (< 0.01%) persons were lost to follow-up with respect to vital status, and these were censored at date of last contact for inclusion in risk sets. The follow-up questionnaires contained standardized questions on respiratory symptoms (American Thoracic Society 1995) and questions to ascertain lifestyle and housing characteristics pertinent to relative exposure to ambient air pollutants, as well as occupational exposures to dust and fumes and indoor sources of air pollution, including environmental tobacco smoke (ETS).

Several air pollutants were estimated for study participants using the statewide network of monitoring stations maintained by the California Air Resource Board (CARB) (Abbey et al. 1991). Because estimated PM_2.5_ measures were not available on a statewide basis during follow-up, only the 3,769 (2,422 females and 1,347 males) belonging to the airport subcohort (those who lived within an airshed adjacent to one of nine California airports with available visibility measures: Alameda, Bakersfield, Fresno, Long Beach, Los Angeles, Ontario, Sacramento, San Jose, and San Diego) were included in this study. Of these, 530 (*n* = 332 females, *n* = 198 males) were excluded because of a history of CHD, stroke, or diabetes at baseline, leaving 3,239 subjects for analyses.

### Estimation of ambient air pollution concentrations. Estimates of monthly ambient

concentrations of PM_10_, ozone, sulfur dioxide, and NO_2_ were formed for study participants for 1973–1998 using fixed-site monitoring stations maintained by CARB. The detailed methods for estimating ambient air pollutants for study participants are described elsewhere (Abbey et al. 1991, 1995a). Briefly, monthly indices of ambient air pollutant concentrations at 348 monitoring stations throughout California were interpolated to geographic ZIP code centroids according to home and work location histories of study participants. These were cumulated and then averaged over time. Interpolations were restricted to ZIP code centroids within 50 km of a monitoring station and were not allowed to cross barriers to airflow or other topographic obstructions > 250 m above the surrounding terrain. Concentrations of PM_10_ before 1987 were estimated using site- and season-specific regressions based on total suspended particles (TSPs) (Abbey et al. 1995a). Since 1987, directly monitored PM_10_ has been used.

Daily estimates of ambient PM_2.5_ concentration were obtained for 11 airsheds from daily measures of visibility collected at the nine California airports for the years 1973–1998 using regression equations relating PM_2.5_ and visibility. Because of wind patterns, Ontario provided three separate airsheds (East, West, Central). Detailed methods for PM_2.5_ estimation have been described previously (Abbey et al. 1995b). Individual monthly average PM_2.5_ concentrations were calculated as the mean of the daily ambient PM_2.5_ estimates for the airshed in which the participant resided. Any month with PM_2.5_ estimates for > 75% of the days was considered to have valid data.

### Ascertainment of deaths.

Fatal CHD, defined by codes 410–414 of the *International Classification of Diseases*, *9th Revision* (ICD-9) (World Health Organization 1977) as either “definite fatal myocardial infarction” or “other definite fatal CHD,” as underlying or immediate cause of death was used to assess fatal CHD.

Deaths were ascertained through 1998 using record linkage with both the California death certificate files and the National Death Index (Centers for Disease Control and Prevention, National Center for Health Statistics, Atlanta, GA, USA). In addition, our tracing procedures, which included church records, were used (Beeson et al. 1989). Thus, among the airport subcohort free of CHD, stroke, and diabetes at baseline, we identified 1,054 total deaths during follow-up. Death certificates were obtained, and a state-certified nosologist, blinded to the exposure status, coded each death certificate according to the ICD-9 codes.

### Statistical analysis.

Sex-specific comparisons of baseline descriptive information between CHD mortality cases and noncases were made using the Student *t*-test or chi-square test.

Time-dependent Cox proportional-hazards regression modeling was used to study associations between pollutants (PM_2.5_, PM_10–2.5_, PM_10_, O_3_, SO_2_, and NO_2_) and CHD mortality with attained age as the time variable (Greenland 1989). This was further augmented by adding the sandwich variance estimate (Lin 1994) to adjust for correlated observations within each airshed. All 11 air-sheds around the nine airports were included in the model. We also included the airports as dummy variables stratified with the Cox model. Rate ratios were calculated for an increment of 10 μg/m^3^ for each of the particulate pollutants and 10 ppb for each of gaseous pollutants, except SO_2_, which was calculated for an increment of 1 ppb. Because measures for most of the pollutants were available only from 1973, we had 4-year monthly averages for these pollutants at baseline in 1977. To standardize the exposure window preceding events, we therefore selected 4-year average as our moving time period of exposure, but excluded the last month before the event to avoid measuring short-term effects. Participants who did not die were censored at end of follow-up, or at time of last contact if they were lost to follow-up (394 females, 166 males). The different pollutants were entered into the model as continuous variables.

**Figure 1:**
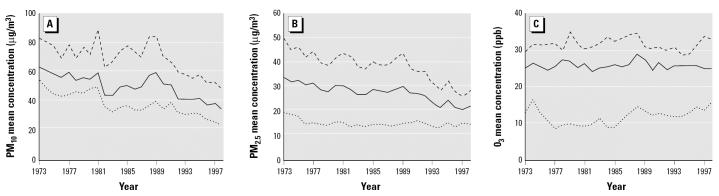
Mean concentration over time, 1973–1998: (*A*) PM_2.5_; (*B*) PM_10_; and (*C*) O_3_. (*A* and *B*) Sexes combined: AHSMOG cohort (solid line), Ontario East air basin (dashed line), and San Diego air basin (dotted line). (*C*) AHSMOG cohort (solid line), mountain areas (dashed line), and coastal areas (dotted line). The *y*-axis scales differ among the three panels.

The basic multivariable model included past cigarette smoking, body mass index (BMI), years of education, and frequency of meat consumption. We added an interaction term between sex and pollutant to this basic model that was significant, and therefore, all analyses were sex specific. Additional candidate variables for inclusion in the final model were ETS (years lived or worked with a smoker), total physical activity at baseline, history of hypertension at baseline, exposure to dust/fumes at work, frequency of eating nuts (Fraser et al. 1992), number of glasses of water per day (Chan et al. 2002), time spent outdoors, and hormone replacement therapy (HRT) (female models). In addition, we found that the levels of PM pollutants used in this study have declined from 1973 to 1998 (Figure 1), and we therefore included calendar time as a candidate variable to adjust for possible changes in PM composition over time. All candidate variables were entered into the basic multivariable model one at a time to assess their impact on the main effect. Only calendar year changed the relative risks (RRs) > 10% (actually 16%) and was retained in the final model (Greenland 1989).

The proportional hazards assumption was checked by examining log [−log(survival)] curves versus the time (attained age) as well as the product term of each respective variable in the final model with the log of the time variable (Greenland 1989). Each of these interaction terms produced a *p*-value > 0.05 based on the Wald statistic, indicating that the proportional hazards assumptions were not seriously violated. This was supported further by visual inspection.

The same sex-specific, time-dependent multivariable Cox proportional-hazards regression models with and without the sandwich variance estimate, airport dummy variables, and stratified analysis were further used to study associations in two-pollutant models for particulates (PM_2.5_, PM_10–2.5_, or PM_10_) with each of the gases (O_3_, SO_2_, and NO_2_) and CHD mortality. We evaluated the interactions between two individual pollutants for inclusion in the final model based on whether they changed the RRs > 10%. None of the terms met this criterion (Greenland 1989). All analyses were repeated for postmenopausal females separately.

In addition, we repeated sex-specific analyses using cumulative monthly averages of each particulate pollutant from 1973 to censoring and also for each of the PM fractions using three levels of exposure (≤25, > 25–38, > 38 μg/m^3^) rather than as a continuous variable. We used the SAS statistical package (version 9.1; SAS institute, Cary, NC) for all analyses.

## Results

During 22-year follow-up (1977–1998), there were 155 CHD deaths in females and 95 among males, 23.7% of all deaths in this group.

**Figure 2:**
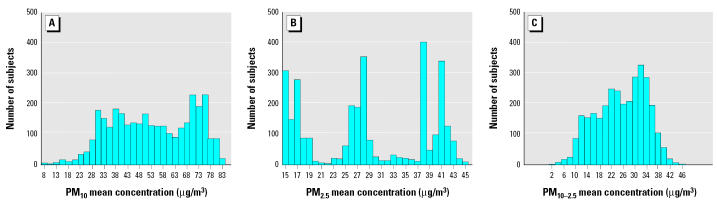
Frequency distribution of mean ambient concentration of (*A*) PM_10_, (*B*) PM_2.5_ , and (*C*) PM_10–2.5_, 1973 to censoring month; *n* = 3,239. Note that the *x*-axis scales differ among the three panels.

Those who died of CHD were older at baseline, had fewer years of education, and were more likely to have hypertension; a larger proportion of the females were postmenopausal, and of these, fewer had used HRT (Table 1). A higher proportion of female noncases had lived or worked with a smoker (ETS), and noncases tended to drink more water than did cases. The mean concentrations and correlations of pollutants for this airport subcohort from 1973 through the month of censoring are provided in Table 2. Frequency histograms of the individual mean ambient concentrations of each of the PM fractions from 1973 to censoring month are given in Figure 2. Those in the lowest distribution of PM_2.5_ lived in the airsheds represented by the San Diego, San Jose, Sacramento, and Alameda airports; medium levels were found in Fresno, Los Angeles International, Bakersfield, Long Beach, Ontario West, and Ontario Central; and the highest distribution represents Ontario East. Figure 1 shows the secular trends in PM_10_, PM_2.5_, and O_3_ during the study for the Ontario East and San Diego air basins and for the study population as a whole.

### Risk of fatal CHD.

All results presented are from the time-dependent Cox model without and with the inclusion of the sandwich variance estimate. For females, in age-adjusted single-pollutant models, a positive but nonsignificant relationship was found between each of the three PM fractions and risk of fatal CHD (Table 3). This association became stronger in multivariate analyses, with PM_2.5_ having the highest RR of 1.42 [95% confidence interval (CI), 1.11–1.81] for each increment of 10 μg/m^3^.

In two-pollutant models with O_3_ (Table 4), the associations with each of the PM fractions became stronger and statistically significant both in age-adjusted and in multivariable-adjusted models, with the strongest relationship for PM_2.5_ (RR = 1.99; 95% CI, 1.37–2.88). NO_2_ did not change the associations between PM and fatal CHD, whereas SO_2_ strengthened the association some, but not to the same degree as did O_3_. Point estimates remained virtually unchanged both in single-pollutant and in multipollutant models when including the sandwich variance estimate. When airports were included as dummy variables or in stratified analyses, the risk estimates either remained the same or were strengthened. Limiting the analyses to postmenopausal females resulted in small increases in risk estimates.

**Figure 3:**
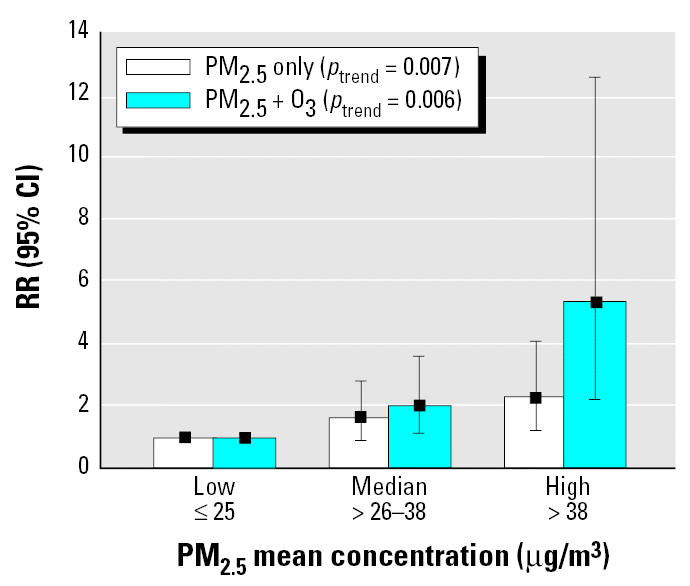
RR of fatal CHD and tertiles of PM_2.5_ mean concentration in single- and two-pollutant models (PM_2.5_ + O_3_); all females.

Using cumulative monthly averages from 1973 to censoring instead of the 4-year moving average gave similar but somewhat weaker associations. Using PM_2.5_ estimates as tertiles (Figure 3 for females) showed that those exposed to levels > 38 μg/m^3^ were 2.3 times more likely to die of CHD than were those living in areas where concentrations were ≤ 25 μg/m^3^ (*p*-value for trend = 0.007). After adjusting for O_3_ in two-pollutant models, the risk estimates for PM_2.5_ increased to 2.03 and 5.35 in the medium and highest tertiles, respectively (*p*-value for trend = 0.006).

No significant associations were found between any of the gaseous pollutants and fatal CHD in either the age-adjusted or multivariable-adjusted analyses in single-pollutant or in two-pollutant models with PM. However, the association with NO_2_ was elevated for both males and females in single-pollutant models (Table 3). In males, no association was found between particulate pollutants and fatal CHD either as continuous or as categorical (tertiles) variables in single- or two-pollutant models (Tables 3, 4).

## Discussion

Most studies of the association between ambient particulate air pollution and cardiovascular disease (CVD) have been limited to effects of short-term increases in PM on hospital admissions for CVD (Zanobetti et al. 2000) and total mortality (Dominici et al. 2003; Samet et al. 2000). Of the particulate pollutants, PM_2.5_ seems to show the strongest association with CVD outcomes (Pope et al. 2002, 2004a).

The Six Cities and the ACS studies have reported a positive association between CPD and cardiovascular deaths and long-term exposure to ambient PM. The association was strongest for fine particles, with RRs varying between 1.06 for CPD deaths (Pope et al. 2002) and 1.12 for cardiovascular deaths (Pope et al. 2004a) for each increment of 10 μg/m^3^ after adjusting for age, sex, diet, and other demographic covariates. When comparing most-polluted with least-polluted areas, the RR for CPD death was 1.31 for a difference of 24.5 μg/m^3^ in the ACS study (Pope et al. 1995) and 1.37 for a difference of 18.6 μg/m^3^ in the Six Cities Study (Dockery et al. 1993). Pope et al. (2004a) reported a somewhat higher risk estimate for mortality from IHD, with an RR of 1.18 for an increment of 10 μg/m^3^, and concluded that “predominant PM mortality associations” were with IHD. The effect of fine particles on CPD mortality has not been reported from AHSMOG to date. For PM_10_ and CPD mortality, no significant relationships were found, but males had higher estimates than did females (Abbey et al. 1999).

Two European cohort studies have both looked at traffic-related pollution (Hoek et al. 2002; Nafstad et al. 2004). Hoek et al. (2002) found that persons living near a major road had a 1.95 greater risk of CPD death than did others and, that for each increase of 10 μg/m^3^ in black smoke, the RR increased by 34%. Among Norwegian men, Nafstad et al. (2004) found that for each increase of 10 μg/m^3^ in nitrogen oxides (markers of traffic pollution), the risk increased by 8% for fatal IHD and by 16% for respiratory deaths.

We found significant relationships between ambient PM and fatal CHD only in females. To our knowledge, no other cohort study on the health effects of ambient air pollution has reported sex-specific risks for CHD mortality. Therefore, we cannot readily compare our findings with others. However, the ACS study did find a slightly higher, although not significant, risk of CPD mortality among never-smoking females versus males in the most-polluted cities compared with the least polluted (RR = 1.57 in females vs. 1.24 in males) (Pope et al. 1995). As far as we have been able to assess, neither the Six Cities Study nor the Dutch study (Hoek et al. 2002) has reported sex-specific findings on CPD mortality. The Norwegian cohort included only males (Nafstad et al. 2004), as did the male U.S. veterans cohort mortality study (Lipfert et al. 2000). In a study of short-term effects, Peters et al. (1997) reported a stronger effect of TSPs on blood viscosity in females than males during episodes of high air pollution in Augsburg, Germany.

Several experimental studies of pulmonary deposition of inhaled particles in healthy adults showed that particle deposition characteristics differ between males and females under controlled breathing conditions. Kim and Hu (1998) found that deposition in females is greater than that in males and that the deposition was more localized within the lung in females. The authors suggest that regional deposition enhancement in women may lead to a greater health risk in females than in males. This is consistent with the hypothesized mechanism in which the deposition of particles in the lung could elicit inflammatory responses resulting in a systemic signal (Seaton et al. 1995).

An experimental study of 50 persons (Sorensen et al. 2003) showed significant positive associations between personal PM_2.5_ exposure and oxidation products [e.g., plasma malondialdehyde, red blood cells (RBCs), and hemoglobin concentrations] in females but not in males. The authors suggest that females possibly are more sensitive to airborne pollution than are males because they have fewer RBCs and thus may be more sensitive to toxicologic influences of air pollutants.

A recent study supporting our sex-differential findings assessed the relationship between ambient levels of PM_2.5_ at place of residence and degree of intima media thickness as measured by ultrasound (Künzli et al. 2005). Cross-sectional analyses of baseline data from two clinical trials in Los Angeles showed that the association was statistically significant among women but not among men. Also, the associations were stronger among older persons who had never smoked or who reported using lipid-lowering treatment at baseline. The strongest association, however, was found among older women (≥60 years of age). These findings corroborate with our findings from AHSMOG, which is also an older population, with mean age at fatal CHD of 67.6 years in men and 72.3 years in women.

Our findings and those of other studies show that particulate air pollution seems to have a stronger effect on fatal CHD than on other fatal CPD end points. The ACS study found a somewhat higher RR associated with an increase in PM_2.5_ of 10 μg/m3 for fatal IHD (RR = 1.18; 95% CI, 1.14–1.23) (Pope et al. 2004a) than what they had previously found for CPD mortality (RR = 1.09; 95% CI, 1.03–1.16) (Pope et al. 2002). In females, our findings for fatal CHD and PM are stronger than those we have previously reported for CPD mortality in the total AHSMOG cohort (Abbey et al. 1999) and in the airport cohort (McDonnell et al. 2000). Also, in a previous report we found positive associations with CPD mortality only in males (Abbey et al. 1999). In extended follow-up of CPD mortality in the total AHSMOG cohort through 1998 using the same models as previously, we continue to find a slightly stronger association in males than in females (unpublished data). However, when we exclude baseline CHD, stroke, and diabetes, these sex differences disappear, and when we limit our analyses to the airport cohort, CPD mortality is actually significantly increased in females but not in males (RR = 1.14 vs. 1.02 in males). These findings warrant further study of the effect of PM in sensitive subgroups and in densely populated areas (e.g., airport cohort) versus less densely populated areas. It also suggests that health effects of air pollution are different in males and females.

Even though we found the strongest association with PM_2.5_, the coarse fraction was also associated with significant risk. One possible explanation for the higher risk estimates for all three PM fractions in our study could be more precise estimates of ambient air pollution and thus less exposure misclassification. AHSMOG is the only study with monthly estimates of ambient air pollution for each subject throughout the entire follow-up period. Other reasons could be the homogeneity of the population (see “Strengths and limitations,” below).

Because different components of air pollution frequently occur together and are highly correlated (Table 2), the U.S. Environmental Protection Agency (EPA) has suggested that the association observed with PM could instead be due to gaseous pollutants (U.S. EPA 1989). We found no significant association between fatal CHD and gaseous pollutants in single- or two-pollutant models. However, in two-pollutant models, both O_3_ and SO_2_ strengthened the relationship between PM and fatal CHD, whereas NO_2_ had no effect. The modifying effect of O _3_ can possibly be explained by findings indicating that lung epithelial permeability increases with exposure to O_3_ (Blomberg et al. 2003), thus making the body more susceptible to intrusion of particulate matter. The proposed mechanisms for the observed cardiovascular effects of particulates have been discussed in detail in a statement from the American Heart Association (Brook et al. 2004). Several pathways may be involved, but initiation of pulmonary and systemic oxidative stress and inflammation by components of the different PM particles seems to be the most accepted. The resulting cascades of physiologic responses are believed to be able to jointly initiate processes that ultimately lead to a CHD event. Elevated ambient PM_2.5_ levels have been shown to be associated with cardiac autonomic function (Peters et al. 2000), heart rate and heart rate variability (Pope et al. 2004b), CRP levels (Riediker et al. 2004), and changes in blood viscosity favoring coagulation (Peters et al. 1997; Seaton et al. 1995). Several authors have suggested that risk of CVD may be mediated, at least partly, through increased concentrations of plasma fibrinogen, possibly due to an inflammatory reaction caused by air pollution (Koenig et al. 1998). Fibrinogen is an important determinant of plasma viscosity and an independent risk factor for CHD (Koenig et al. 1998). Numerous animal models corroborate the findings in humans of an effect of PM on heart rate (Chang et al. 2004), blood viscosity (Coates and Richardson 1978), and pulmonary inflammation (Wichers et al. 2004).

These pathways are very similar to those suggested for the effect of cigarette smoking on risk of CHD, such as elevated inflammatory markers, especially CRP levels (Panagiotakos et al. 2004), fibrinogen and white cell counts (Panagiotakos et al. 2004), blood viscosity (Frohlich et al. 2003), heart rate (Bolinder and de Faire 1998), and oxidative stress (Guthikonda et al. 2004). Smoking also has been found to trigger acute vasoconstriction and thus the enhanced development of atherosclerosis in the systemic vasculature (Kiechl et al. 2002). Finally, in studies of the effect of smoking and ETS, Diez-Roux et al. (1995) and Howard et al. (1994) have reported clear effects on intima media thickness progression over time and on arterial wall stiffness (Mack et al. 2003).

### Strengths and limitations.

Because all subjects in AHSMOG are nonsmokers, our results are free from the confounding of active cigarette smoking. We had detailed information about ETS and have been able to adjust for this effect. Any modifying effect of alcohol is also eliminated because virtually everyone abstains from alcohol. Because AHSMOG has extensive information on lifestyle, we were able to adjust for the effects of a number of such factors, including dietary factors, found to be associated with CHD in this cohort. This adjustment actually strengthened the associations between PM and fatal CHD in females but not in males.

Although we have shown cardiovascular effects of particulate air pollution in this study, we have unknown amounts of measurement error in both the estimated long-term ambient concentrations of pollutants and other covariates. One source of measurement error derives from interpolating ambient concentrations (PM_10_, O_3_, NO_2_, SO_2_) from fixed-site monitoring stations to ZIP code centroids of work and home locations of study participants (Abbey et al. 1991, 1995a). Another source of measurement error is that ambient PM_2.5_ concentration was not measured directly for the duration of this study, but estimated from airport visibility, temperature, and humidity (Abbey et al. 1995b). The precision of the PM_10–2.5_ is unknown because it is calculated as the difference between PM_10_ and PM_2.5_. Use of ambient concentrations rather than measures of personal exposure could be one limitation in this study, but it is unlikely that we have selective bias in the females only. Further, we cannot rule out the possibility that the observed sex difference in effect could be due to measurement error. Males, more than females, reported working > 5 miles from their residence and thus may have spent more time in heavy traffic (more commutes and longer commuter distances). We have not been able to take this into consideration when estimating each subject’s ambient air pollution levels.

## Conclusions

In summary, in this study we found an elevated risk of fatal CHD associated with ambient levels of PM_10_, PM_10–2.5_, and PM_2.5_ in females but not in males. The risk estimates were strengthened when adjusting for gaseous pollutants and were highest for PM_2.5_. Our findings are in line with findings by others of an effect of PM on CPD mortality, but are of greater magnitude, possibly because the outcome was limited to fatal CHD with better control of confounding factors such as alcohol and tobacco.

Further studies are needed from larger cohorts and/or with longer follow-up to support our findings of a sex-differential effect of PM on risk of fatal CHD. Developing more accurate ways to assess an individual’s exposure to ambient levels of PM will improve precision of risk estimates. Further, it is important to study whether the effects of air pollution are reversible in a manner similar to that found when smokers stop smoking. The effect of different exceedance frequencies should also be explored as well as the effect of different chemical compositions of PM.

## Correction

Some of the values in Table 3 published originally online were incorrect; they have been corrected here.

## Figures and Tables

**Table 1:** Selected characteristics of study population at baseline.

	Male (*n* = 1,149)		Female (*n* = 2,090)	
Characteristic	Cases (*n* = 95)	Noncases (*n* = 1,054)	Cases (*n* = 155)	Noncases (*n* = 1,935)
Age [years (mean ± SD)]	67.6 ± 11.5	55.8 ± 12.9**	72.3 ± 8.9	56.6 ± 13.4**
Years of education (mean ± SD)	13.5 ± 3.5	14.6 ± 3.2*	12.6 ± 2.8	13.4 ± 2.6**
Never smokers	51 (53.7)	717 (68.0)*	133 (85.8)	1,655 (85.5)
BMI at or above median	46 (48.4)	477 (45.3)	76 (49.0)	875 (45.2)
Meat consumption^a^^,^^b^				
< 1 week	40 (42.1)	496 (47.1)	88 (56.8)	913 (47.2)
1 week	50 (52.6)	516 (49.0)	57 (36.8)	917 (47.4)
Total exercise				
Low	25 (26.3)	344 (32.6)	67 (43.2)	937 (48.4)
Moderate and high	70 (73.7)	709 (67.3)	83 (53.5)	990 (51.2)
History of hypertension	32 (33.7)	171 (16.2)**	70 (45.2)	444 (22.9)**
ETS	57 (60.0)	619 (58.7)	77 (49.7)	1,208 (62.5)*
Nuts^a^				
≤2/month	29 (30.5)	331 (31.4)	60 (38.7)	684 (35.3)
1–4/week	37 (38.9)	428 (40.6)	51 (32.9)	736 (38.0)
≥5/week	23 (24.2)	255 (24.2)	33 (21.3)	397 (20.5)
Water^a^^,^^c^				
≤2 glasses	6 (6.3)	119 (11.3)	26 (16.8)	351 (18.1)
3–4 glasses	44 (46.3)	369 (35.0)	49 (31.6)	708 (36.6)
≥5 glasses	42 (44.2)	546 (51.8)	79 (51.0)	833 (43.0)
Postmenopausal			138 (89.0)	1,323 (68.4)**
HRT in postmenopausal females			20 (14.5)	431 (32.6)**

Values are presented as no. (%) or mean ± SD.

a Some columns do not add to 100% because of missing data.

b Significant at *p* < 0.01 for females only.

c Significant at *p* < 0.05 for males only.

* *p* < 0.01,

** *p* < 0.001.

**Table 2:** Descriptive statistics and correlations between long-term averages of pollutants estimated for study participants, 1973 through month of censoring, females and males combined (*n* = 3,239).

	PM_10_ (μg/m^3^)	PM_2.5_ (μg/m^3^)	PM_10–2.5_ (μg/m^3^)	O_3_ (ppb)	NO_2_ (ppb)	SO_2_ (ppb)
Mean ± SD	52.6 ± 16.9	29.0 ± 9.8	25.4 ± 8.5	26.2 ± 7.3	34.9 ± 9.7	4.5 ± 2.7
PM_10_	1.00	0.83*	0.91*	0.79*	0.50*	0.36*
PM_2.5_		1.00	0.59*	0.60*	0.25*	0.30*
PM_10–2.5_			1.00	0.75	0.51*	0.35*
O_3_				1.00	0.22*	0.11*
NO_2_					1.00	0.70*
SO_2_						1.00

* *p* < 0.01.

**Table 3:** Age-adjusted and multivariable-adjusted RRs of fatal CHD for specific PM components: single-pollutant models.

			Age adjusted		Multivariable adjusted^a^		Multivariable adjusted^b^		Postmenopausal females, multivariable adjusted^b^	
	Pollutant	Increment	Cases	RR (95% CI)	Cases	RR (95% CI)	Cases	RR (95% CI)	Cases	RR (95% CI)
Females	PM_10_	10 μg/m^3^	92	1.11 (0.98–1.26)	92	1.22 (1.06–1.40)	92	1.22 (1.01–1.47)	80	1.30 (1.08–1.57)
	PM_2.5_	10 μg/m^3^	92	1.19 (0.96–1.47)	92	1.42 (1.11–1.81)	92	1.42 (1.06–1.90)	80	1.49 (1.17–1.89)
	PM_10–2.5_	10 μg/m^3^	92	1.20 (0.95–1.53)	92	1.38 (1.07–1.77)	92	1.38 (0.97–1.95)	80	1.61 (1.12–2.33)
	O_3_	10 ppb	92	0.89 (0.67–1.18)	92	0.97 (0.71–1.32)	92	0.97 (0.68–1.38)	80	1.07 (0.73–1.59)
	NO_2_	10 ppb	92	1.09 (0.88–1.35)	92	1.17 (0.92–1.49)	92	1.17 (0.98–1.40)	80	1.20 (1.01–1.44)
	SO_2_	1 ppb	87	0.93 (0.87–1.01)	87	0.94 (0.85–1.04)	87	0.94 (0.81–1.08)	77	0.94 (0.80–1.11)
Males	PM_10_	10 μg/m^3^	53	0.95 (0.81–1.11)	53	0.94 (0.80–1.11)	53	0.94 (0.82–1.08)		
	PM_2.5_	10 μg/m^3^	53	0.89 (0.69–1.17)	53	0.90 (0.67–1.19)	53	0.90 (0.76–1.05)		
	PM_10–2.5_	10 μg/m^3^	53	0.93 (0.68–1.29)	53	0.92 (0.67–1.28)	53	0.92 (0.66–1.29)		
	O_3_	10 ppb	53	0.87 (0.58–1.29)	53	0.89 (0.59–1.33)	53	0.89 (0.60–1.30)		
	NO_2_	10 ppb	53	1.24 (0.94–1.64)	53	1.16 (0.86–1.56)	53	1.16 (0.89–1.51)		
	SO_2_	1 ppb	51	1.06 (0.98–1.14)	51	1.02 (0.92–1.13)	51	1.02 (0.94–1.11)		

a Adjusted for smoking status (past vs. never), years of education, BMI (below vs. at or above median), meat consumption (< 1/week vs. ≥1/week), calendar time.

b Model “b” with sandwich variance estimate.

**Table 4:** Age-adjusted and multivariable-adjusted RRs of fatal CHD for specific PM components: two-pollutant models.

			Age adjusted^a^		Multivariable adjusted^b^		Multivariable adjusted^c^		Postmenopausal females, multivariable adjusted^c^	
	Pollutant PM	Gas	Cases	RR^d^ (95% CI)	Cases	RR^d^ (95% CI)	Cases	RR^d^ (95% CI)	Cases	RR^d^ (95% CI)
Females	PM_10_ +	O_3_	92	1.33 (1.12–1.59)	92	1.45 (1.21–1.74)	92	1.45 (1.31–1.61)	80	1.52 (1.37–1.69)
		NO_2_	92	1.11 (0.97–1.26)	92	1.21 (1.05–1.40)	92	1.21 (1.00–1.46)	80	1.29 (1.06–1.57)
		SO_2_	87	1.15 (1.02–1.31)	87	1.27 (1.10–1.47)	87	1.27 (1.08–1.50)	77	1.33 (1.11–1.59)
	PM_2.5_ +	O_3_	92	1.61 (1.17–2.22)	92	1.99 (1.37–2.88)	92	2.00 (1.51–2.64)	80	1.95 (1.52–2.50)
		NO_2_	92	1.18 (0.95–1.47)	92	1.39 (1.08–1.80)	92	1.40 (1.04–1.87)	80	1.46 (1.13–1.89)
		SO_2_	87	1.36 (1.05–1.74)	87	1.50 (1.15–1.97)	87	1.51 (1.17–1.95)	77	1.51 (1.19–1.92)
	PM_10–2.5_ +	O_3_	92	1.47 (1.10–1.96)	92	1.62 (1.21–2.17)	92	1.62 (1.31–2.01)	80	1.85 (1.50–2.29)
		NO_2_	92	1.19 (0.92–1.54)	92	1.35 (1.03–1.76)	92	1.34 (0.94–1.94)	80	1.59 (1.07–2.36)
		SO_2_	87	1.31 (1.03–1.68)	87	1.49 (1.15–1.93)	87	1.49 (1.12–1.99)	77	1.68 (1.20–2.35)
Males	PM_10_ +	O_3_	53	0.97 (0.78–1.20)	53	0.96 (0.77–1.19)	53	0.96 (0.87–1.05)		
		NO_2_	53	0.90 (0.76–1.07)	53	0.91 (0.76–1.09)	53	0.91 (0.78–1.07)		
		SO_2_	51	0.92 (0.78–1.09)	51	0.93 (0.78–1.11)	51	0.93 (0.78–1.11)		
	PM_2.5_ +	O_3_	53	0.92 (0.65–1.29)	53	0.91 (0.64–1.30)	53	0.91 (0.78–1.06)		
		NO_2_	53	0.82 (0.61–1.10)	53	0.85 (0.63–1.15)	53	0.85 (0.70–1.04)		
		SO_2_	51	0.86 (0.65–1.14)	51	0.88 (0.65–1.19)	51	0.88 (0.73–1.07)		
	PM_10–2.5_ +	O_3_	53	1.01 (0.67–1.51)	53	0.97 (0.64–1.46)	53	0.97 (0.74–1.26)		
		NO_2_	53	0.86 (0.62–1.20)	53	0.87 (0.62–1.23)	53	0.87 (0.60–1.26)		
		SO_2_	51	0.90 (0.64–1.27)	51	0.89 (0.63–1.27)	51	0.85 (0.55–1.32)		

a Age adjusted with sandwich variance estimate.

b Adjusted for smoking status (past vs. never), years of education, BMI (below vs. at or above median), meat consumption (< 1/week vs. ≥1/week), calendar time.

c Model “b” with sandwich variance estimate.

d RR was calculated for an increase of 10 μg/m^3^ in concentration of the specific PM components.
